# Supplementary material for: Phenomenon of music-induced opening of the blood-brain barrier in healthy mice
Source: Proc Biol Sci. 2020 Dec 16;287(1941):20202337. doi: 10.1098/rspb.2020.2337 (PMC7779516; doi:10.1098/rspb.2020.2337)
Supplement: Supplementary Figures, Table and Material and Methods [file rspb20202337supp1.pdf]

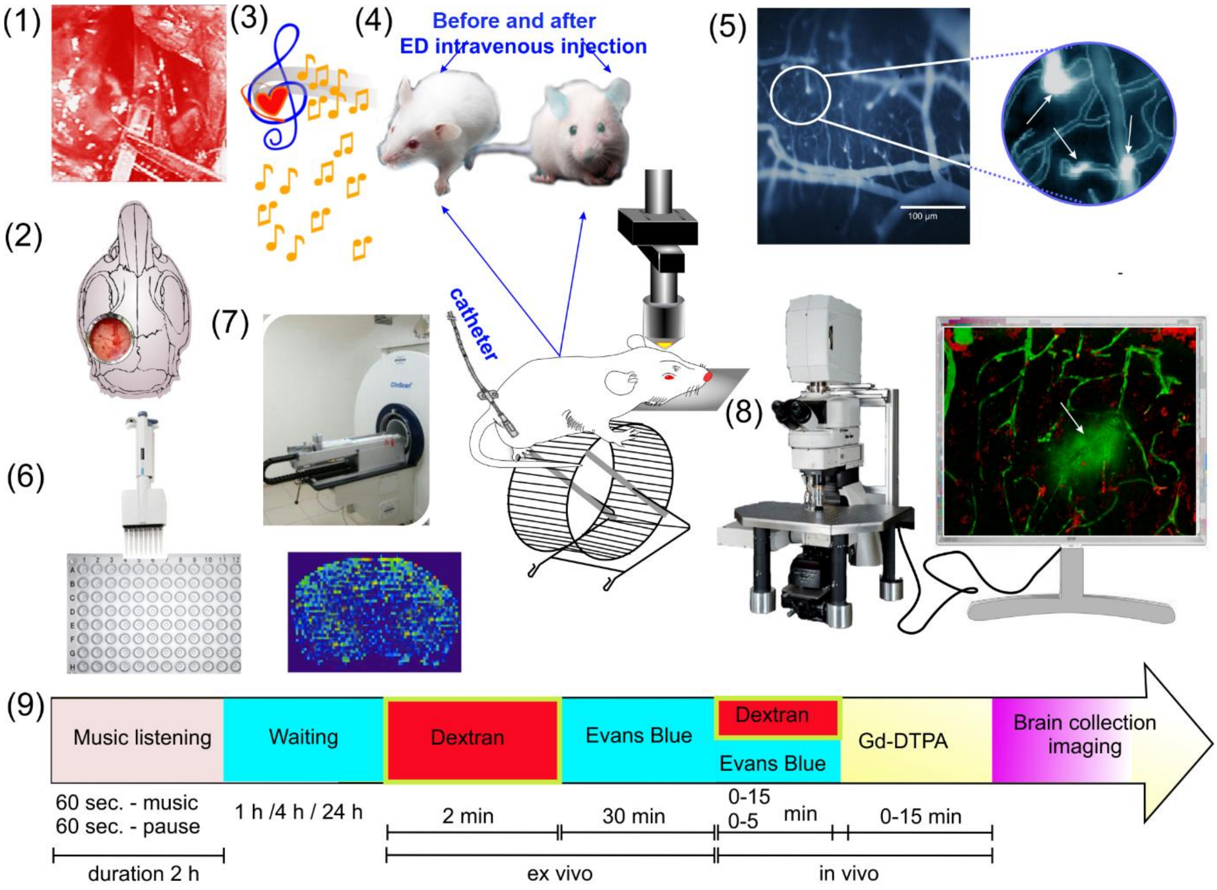


**Fig. 1 SI. Design of *in vivo* and *ex vivo* experiments of the study of loud music-induced opening of the blood-brain barrier (OBBB)**: (1) three days before experiments the polyethylene catheter (PE-10 tip, Scientific Commodities Inc., Lake Havasu City, Arizona) was implanted into the femoral vein for injection of tracers (Evans Blue dye, fluorescein isothiocyanate dextran 70 kDa, gadolinium-diethylene-triamine-pentaacetic acid - Gd-DTPA); (2) – the optical window [1] was prepared for *in vivo* real time fluorescent microscopy of the BBB permeability to the Evans Blue Albumin Complex (EBAC, 68.5 kDa) in awake behavior mice (see session "In vivo real time fluorescent microscopy of extravasation of Evans Blue"); (3) – afterward mice (n=15 in each group) were underwent to the intermittent music (70-90-100 dB, 11-10,000 Hz, Scorpions "Still loving you") during 2h (60 sec sound and 60 sec – pause) (see session "Experimental design of music effect on the BBB permeability"); (4) – Evans Blue dye was injected via catheter immediately after music-off and then we performed *in vivo* real time fluorescent microscopy of OBBB for EBAC in awake behavior mice during 5 hrs; (5) The EBAC leakage was detected as bright fluorescence around the cerebral microvessels; (6) after *in vivo* real time fluorescent microscopy of OBBB for EBAC, all mice were decapitated, their brain removed and analyzed using spectrofluorometric assay of EBAC extravasation (see session "Spectrofluorometric assay of EBAC extravasation"); (7) additionally, magnetic resonance imaging (MRI) was used for the study of the BBB permeability for Gd-DTPA; (8) also *ex vivo* confocal imaging and *in vivo* real-time two-photon laser scanning microscopy of FITC-dextran extravasation was performed; (9) the time points for injections of the model compounds in relation to the music intervention and the brain collection.

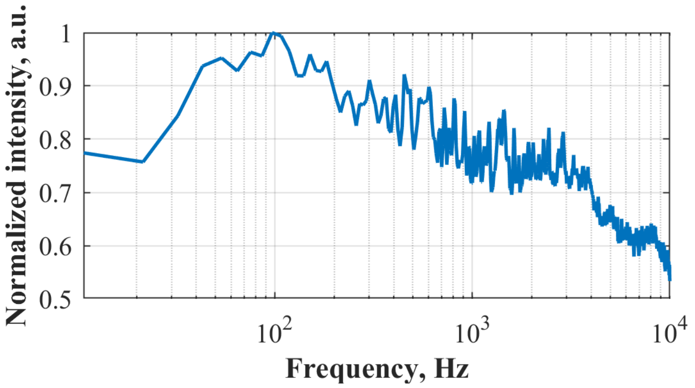


**Fig. 2 SI. The frequency range of music (Scorpions "Still Loving You")**: frequencies in the range of 11-10,000 Hz and maximal intensity around 100 Hz.

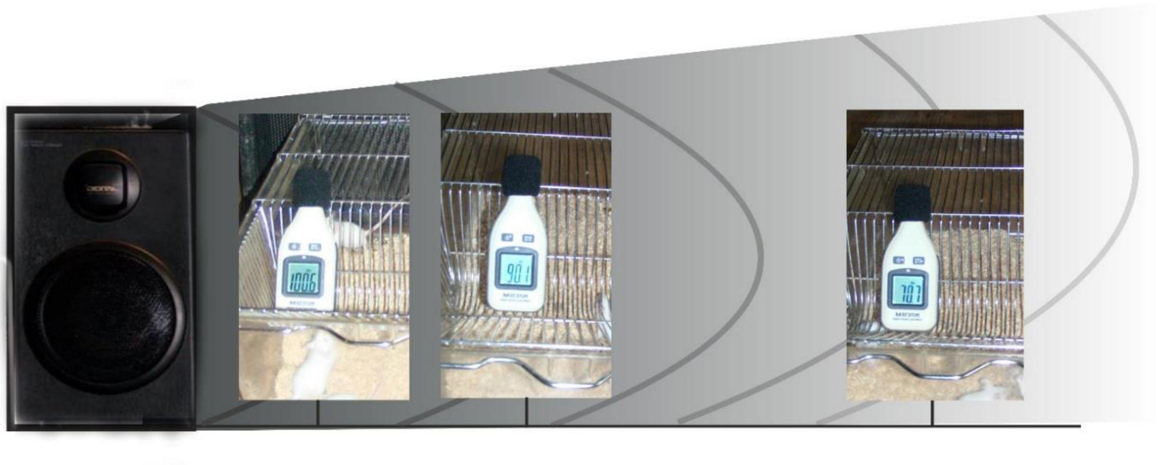

**Fig. 3 SI. The measure of sound level in the cages of animals during listening of loud music.** To produce the loud music, we used loudspeaker generated sound 100 dB. The location of cage with mice from loudspeaker was: 1) at 1m - 100 dB; 2) at 2m - 90 dB; 3) at 32m - 70 dB in according with information referred here [https://lgmproducts.com/technical-information/sound-pressure-levels-in-dba]. The sound energy was measured directly in a cage of animals using a sound level meter (Megeon 92130, Russia).

# Table 1 SI. The effect of loud music on the BBB permeability to EBAC (μg/g tissue)

| **Sound level (dB) and time elapsed after sound exposure (h)** | **Content of EBAC (μg/g tissue)** | | |
|---|---|---|---|
| No music (the control group) | 0.15±0.01 | | |
| | **Music** duration during 2 h (intermittent) | **Music** duration during 1 h (intermittent) | **Music** duration during 0.25 h (continues) |
| 100 dB | | | |
| immediately | 0.12±0.01 | 0.11±0.05 | 0.14±0.02 |
| ¼ h | 0.10±0.05 | 0.14±0.01 | 0.17±0.06 |
| ½ h | 0.13±0.03 | 0.12±0.05 | 0.12±0.02 |
| 1 h | **2.60±0.06 ***** | 0.12±0.03 | 0.17±0.01 |
| 4 h | 0.19±0.03 | 0.15±0.07 | 0.15±0.05 |
| 24 h | 0.16±0.03 | 0.11±0.09 | 0.17±0.02 |
| 90 dB | | | |
| immediately | 0.15±0.08 | 0.11±0.06 | 0.17±0.01 |
| 1 h | **2.70±0.04** ***(n=11) | 0.16±0.03 | 0.16±0.08 |
| 1 h | 0.18±0.06 (n=4)# | 0.14±0.03 | 0.14±0.05 |
| 4 h | 0.15±0.03 | 0.17±0.02 | 0.18±0.01 |
| 24 h | 0.19±0.07 | | |
| 70 dB | | | |
| immediately | 0.13±0.02 | 0.16±0.07 | 0.12±0.03 |
| 1 h | 0.17±0.08 | 0.11±0.04 | 0.14±0.09 |
| 4 h | 0.19±0.06 | 0.13±0.02 | 0.14±0.02 |
| 24 h | 0.19±0.09 | 0.18±0.02 | 0.11±0.01 |

$p<0.001$: *** - vs. before music exposure (the control group), n=15 for the groups (music duration 2h) and n=10 for the groups (music duration 0.25-1 h); # - the number of mice without the BBB opening.

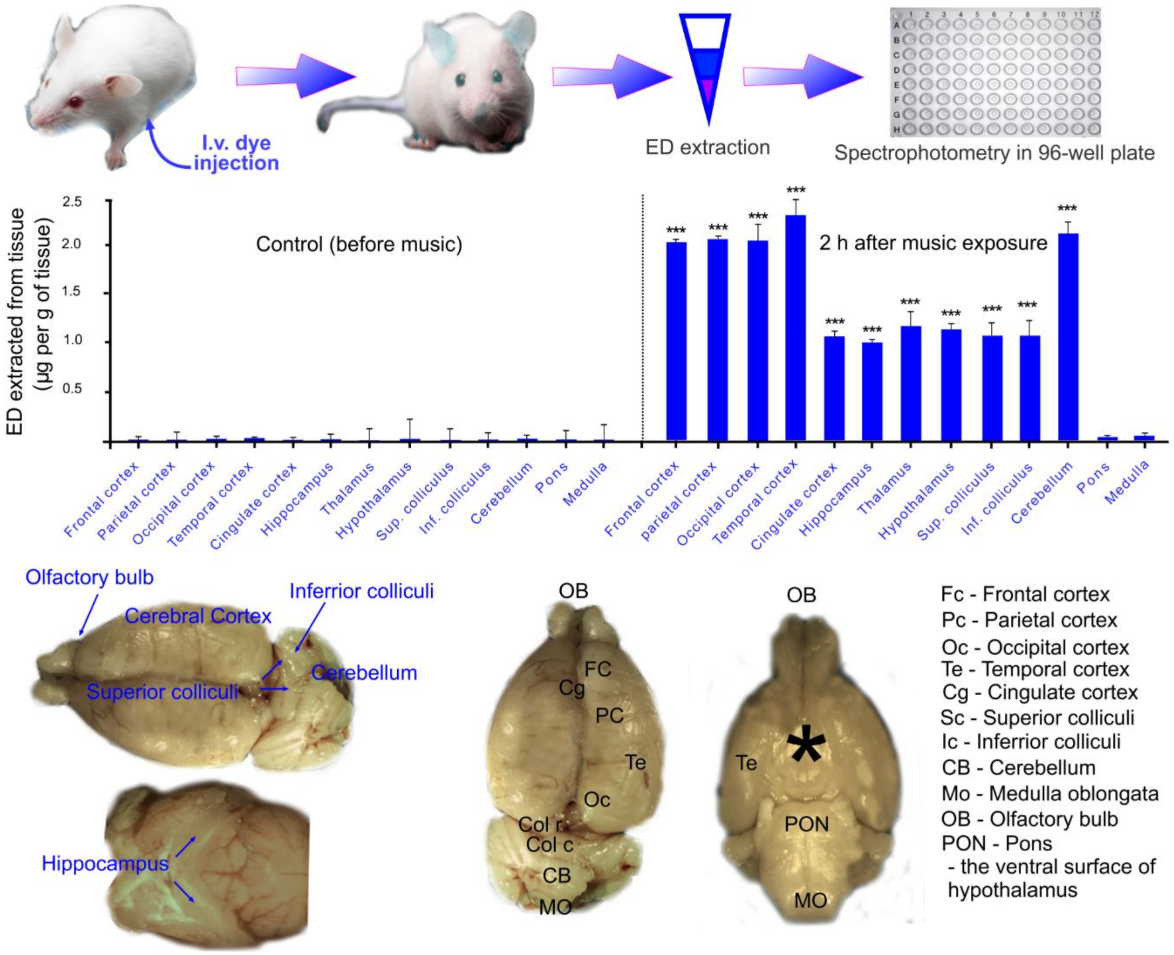


**Fig. 4 SI. The effect of loud music on the BBB permeability to EBAC (μg/g tissue) in different brain fields**: *** - $p<0.001$ vs. the control group (no music), n=15 in each group.

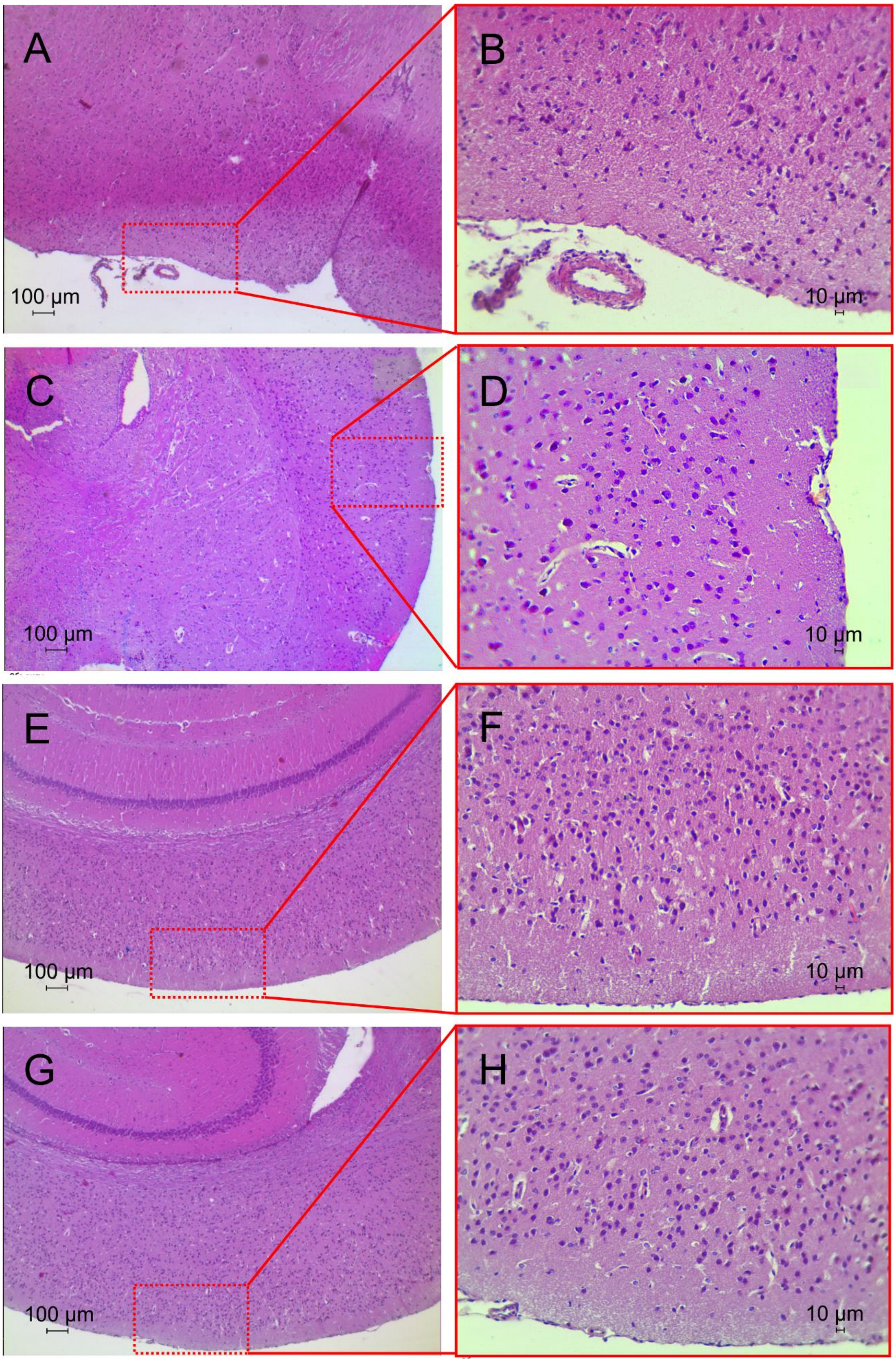


**Fig. 5 SI. Histological analysis of the brain tissues before and after loud music exposure**: A and B – the control group, no music influences, C and D, E and F, G and H – 1h, 4h and 4 weeks after music exposure, respectively, n=10 in each group. Hematoxylin & Eosin staining. Bars represent 10 µm (246.4X).

**Video resources:**

Video 1 illustrates confocal imaging of music-induced opening of BBB to FITC Dextran 70 kDa (arrowed); FITC Dextran – Green (1 mg/25 g mouse, 0.5 % solution in saline, Sigma-Aldrich, i.v.); NG2 (pericyte marker) –Red: https://youtu.be/Gk6uAMcCesg

Video 2 illustrates confocal imaging of the cerebral microvessels in the control group (before music exposure; FITC Dextran – Green (1 mg/25 g mouse, 0.5 % solution in saline, Sigma-Aldrich, i.v.); NG2 (pericyte marker) –Red: https://youtu.be/3CA1yNWdbCA

Video 3 illustrates confocal imaging of the deep cervical lymph node after music-induced opening of BBB to FITC Dextran 70 kDa; FITC Dextran – Green (1 mg/25 g mouse, 0.5 % solution in saline, Sigma-Aldrich, i.v.), Lyve1/Prox1 (markers of the lymphatic endothelium) - Blue/Red: https://youtu.be/PtZb-3nG45s

Video 4 illustrates confocal imaging of the deep cervical lymph node in the control group (before music exposure); FITC Dextran – Green (1 mg/25 g mouse, 0.5 % solution in saline, Sigma-Aldrich, i.v.), Lyve1/Prox1 (markers of the lymphatic endothelium) - Blue/Red: https://youtu.be/Yr8yglqMEzY

## Material and Methods

**Subjects**. The experiments were conducted on male mongrel mice (20-25 mg). All procedures were performed in accordance with the Guide for the Care and Use of Laboratory Animals. The experimental protocols were approved by the Local Bioethics Commission of the Saratov State University (Protocol No. 7) and the Institutional Animal Care and Use Committee of the University of New Mexico, USA (200247).

**Experimental design of music effect on the BBB permeability** is presented in Figure 1 in SI. To produce music (70-90-100 dB and 11-10,000 Hz, Scorpions “Still loving you”) we used loudspeaker (ranging of sound intensity – 0-130 dB, frequencies - 63-15000 Hz; 100 V, Yerasov Music Corporation, Saint Petersburg, Russia) (Figure 2 in SI). The repetitive music exposure was performed using the sequence of: 1 min – music on, then 1 min – music off during 2h. The sound level was measured directly in a cage of animals using the sound level meter (Megeon 92130, Russia) (Figure 3 in SI).

**Spectrofluorometric assay of EBAC extravasation**. The leakage of EBAC was determined in mice in four groups: I) control, no music, II, III and IV) 1, 4 and 24h after music exposure, respectively, n=10-15 in each group (Table 1 and 2 in SI). Before or 1h/4h/24h after music interventions, Evans Blue dye (Sigma Chemical Co., St. Louis, Missouri, 2 mg⁄25 g mouse, 1% solution in physiological 0.9% saline) was injected into the femoral vein and circulated in the blood for 30 min. Then, the mice were decapitated, and their brains were quickly collected. To study the role of auditory system in music-OBBB, the EBAC leakage was evaluated in the intact and deafness mice, n=10 in each group. To exclude the effects of anesthesia on the BBB permeability, the EBAC level was evaluated in the additional group of mice received 2% isoflurane at 1L/min $N_2O/O_2$ – 70:30 during 30 min (the time of duration of anesthesia in *in vivo* experiments), n=10.

***In vivo* real-time fluorescent microscopy of extravasation of Evans Blue** from the cerebral vessels into the brain tissues was performed via optical window using adapted protocol for two-photon imaging of the cortex in awake behavior mice [1]. Fifteen min before imaging, an optical window [2] was prepared in coordinates 1-4 mm caudal and 1-4 mm lateral to bregma. Three days before the experiment, a polyethylene catheter (PE-10 tip, Scientific Commodities Inc.,

Lake Havasu City, Arizona) was inserted into the right femoral vein for Evans Blue intravenous injection in a single bolus dose (2 mg⁄100 g, 1% solution in physiological 0.9% saline). Microscope (Axio Imager A1, Zeiss, Germany) was equipped with CMOS camera (acA1920-40uc, Basler AG, Germany), 10× 0.3 objective lens, and Evans Blue dye filter set 49019 (Chroma, USA). Continuous-wave laser diode module (50000463, Laserlands.net, China) with 160 mW output power at 635 nm was used to excite the dye fluorescence. Laser beam was expanded with a cylindrical lens (f = 50 mm) and then directed towards an object at 45° with respect to the microscope optical axis. To reduce the sample irradiation, the laser was synchronized with the camera "fire" output to turn it on only for the image capturing period. The awake behavior mice were positioned at the microscope stage using 3D printed homemade system for moving paws in a rotating circle and fixation of head. To adapt mice for experimental conditions, they trained to be with fixed head and move paws on a rotating sphere in the microscope system without any performance for two weeks (Figure 1 in SI).

**Confocal microscopy of FITC-dextran 70 kDa extravasation**. The confocal microscopy of the BBB permeability performed in the groups: 1) control, no music; 2) 1h; 3) 4h; 4) 24h after music exposure; n=10 in each group. Before or 1h/4h/24h after music interventions, Fluorescein isothiocyanate (FITC)-dextran 70 kDa (FTICD) (1 mg/25 g mouse, 0.5% solution in saline, Sigma-Aldrich) was injected into the tail vein and allowed to circulate for 2 min. Afterward, mice were decapitated and the brains were quickly removed and fixed in 4% paraformaldehyde (PFA) for 24 h, cut into 50-μm thick slices on a vibratome (Leica VT 1000S Microsystem, Germany) and analyzed using a confocal microscope (Olympus FV10i-W, Olympus, Japan).

**In vivo real-time two-photon laser scanning microscopy (2PLSM).** The BBB permeability via optical window [2] was continuously monitored by measuring the perivascular tissue fluorescence of FITCD 70 kDa (Sigma-Aldrich, in saline 5% wt/vol) in 10 mice at different time points: before or 1, 4 and 24h after music exposure as described previously with some modifications [3]. During the imaging mice were kept under inhalation anesthesia with 2% isoflurane at 1L/min $N_2O/O_2$ – 70:30. FITCD was injected through the tail vein (~100 μl) at an estimated initial concentration in blood serum of 150 μM. The BBB permeability was evaluated by measuring changes in perivascular tissue fluorescence in planar images of the cortex taken 50 and 150 μm depth in 20 min after FITCD injection using Olympus microscope (Japan) [3].

**MRI analysis of the BBB permeability**. **MRI analysis of the BBB permeability**. The MRI with gadolinium-diethylene-triamine-pentaacetic acid (Gd-DTPA, MW= 938 Da; Bayer Healthcare, 0.1 mM/kg) was conducted on the same mice, which we used for 2PLSM, at different time intervals 0 – before and 1, 4, 24h after sound exposure on a 7-T dedicated research MRI scanner (Bruker Biospin; Billerica, MA, USA). Signal transmission and detection was done with a small-bore linear RF coil (inner diameter of 72 mm) and a single tuned surface coil (RAPID Biomedical, Rimpar, Germany). The mice kept under inhalation anesthesia (2% isoflurane at 1L/min $N_2O/O_2$ – 70:30). To non-invasively evaluate the BBB permeability, we used a modified dynamic contrast-enhanced (DCE)-MRI and graphical analysis of the resultant image data [5]. Mice were injected 0.1 mM/kg of gadolinium-diethylene-triamine-pentaacetic acid (Gd-DTPA, MW= 938 Da; Bayer Healthcare) as a bolus injection in the tail vein, followed by imaging. DCE-MRI was performed using a transverse fast T1 mapping that consisted of obtaining pre-contrast (three sequences) and post-contrast (15 sequences) images up to 15 min

after the contrast injection. The details of the pulse sequence T1 RARE for T1 weighted imaging are: FOV = 2 cm × 2 cm, slice thickness = 1.5 mm, slice gap = 0, matrix size = 128 × 128, TR/TE =377 ms/12.3 ms, number of averages = 2, total scan time = 48 s 25 ms.

*MRI Data Analysis and Processing.* The method is based on the leakage of the contrast agent from plasma compartment into brain compartment through BBB resulting in a change of the MR signal intensity. The rate of changes in the MRI signal intensity relates to the BBB permeability (Ki). The T1 map was reconstructed with the t1epia fitting function in the Bruker ParaVision Image Sequence Analysis (ISA) tool. Previous research has demonstrated that the blood-to-tissue transfer or influx constant, $K_i$, could be obtained by a graphical analysis of timed series of tissue and arterial concentrations of a contrast agent [4]. Since the contrast agent concentration is proportional to changes of 1/T1(Δ(1/T1(t))), the color-coded map of $K_i$ was constructed from repeated estimates of Δ(1/T1(t)), where pixels with higher intensity color represent higher BBB permeability. A custom-made computer program in MATLAB (Mathworks, Massachusetts, USA), which implemented the above principle, was used to generate the $K_i$ map.

**Biochemical assays of plasma epinephrine.** The plasma epinephrine level (ng/ml) was determined using ELISA kits (Abnova, Taiwan) before music, immediately, 1h and 4h after music exposure in mice (n=10 in each group). The plates were read at 450 nm using an ELx 800 plate reader (BioTek Instruments Inc.). The detection limits were 0.3 ng/ml (with intra and inter assay variation coefficients of 11.2-16.3% and 8.7-12.6%, respectively).

**Laser speckle contrast imaging of rCBF**. A custom-made laser speckle contrast imaging system was used to monitor rCBF before, immediately and 1h/4h/24h after sound exposure in 10 mice under the inhalation anesthesia (2% isoflurane, 70% $N_2O$ and 30% $O_2$) via an optically cleared skull window (diameter: 5 mm) using optical clearing method FDISCO described in detail in Ref. [2]. The automated segmentation algorithm, described in our previous work [5], was used to calculate the mean value of CBF at macro- (in the Sagittal sinus) and micro-levels.

**Immunohistochemical assay**. Mice in the control group (before music, n=10) and in experimental groups immediately, 1-4-24 hrs after music exposure (n=10 in each group) were euthanized with an intraperitoneal injection of a lethal dose of ketamine and xylazine and intracardially perfused with 0.1 M of PBS for 5 min. Afterward, the brains were removed and fixed in 4% buffered paraformaldehyde for one day and in 20% sucrose for another day. The signal intensity from the examined proteins were evaluated on free-floating sections using the standard method of simultaneously combined staining (Abcam Protocol). Brain slices (50 μm) were blocked in 150 μl 10% BSA/0.2% Triton X-100/PBS for 2 h, then incubated overnight at 4 C and 2 h at room temperature with CLND-5, ZO-1 (1:500; Santa Cruz Biotechnology, Santa Cruz, USA), OCC and NG2 (1:500; Abcam, Cambridge, UK). After several rinses in PBS, the slides were incubated for 3h at room temperature with fluorescent- labeled secondary antibodies on 1% BSA/0.2% Triton X-100 /PBS (1:500; Goat A/Rb, Alexa 555 and 647 Abcam, UK). Confocal microscopy of the cerebral cortex was performed using confocal microscope with water immersion Olympus FV10i-W (Olympus, Japan). In all cases, 10 regions of interest were analyzed.

**Assessment of apoptosis with TUNEL method**. The number of apoptotic cells before and 1h and 4 weeks after music exposure (n=10 in each group) was evaluated with the TUNEL

method using the "17- 141 TUNEL Apoptosis Detection Kit" (Abcam, UK) in accordance with the standard protocol provided by the manufacturer and analyzed by confocal microscopy (Olympus FLUOVIEW FV10i-W, Tokyo, Japan).

For **histological analysis of the brain tissues and SGCs** (see details of embedding of auditory bulla and capsule in SI), three groups of animals were used: I) control group, no music; II and III experimental groups, 1h and 4 weeks after music exposure, respectively; n=10 in each group. All mice were euthanized with an intraperitoneal injection of a lethal dose of ketamine and xylazine. Afterward, the brains were removed and fixed in 10% buffered paraformaldehyde. The paraformaldehyde-fixed specimens were embedded in paraffin, sectioned (4 μm) and stained with hematoxylin and eosin. The histological sections were evaluated by light microscopy using the digital image analysis system Mikrovizor medical μVizo-103(LOMO,Russia).

**Sensorineural deafness model**

To establish an animal model of deafness, we used a synergistic ototoxic effect of a single administration of Furosemide (100 mg/kg, iv, St. Louis, MO, USA) and Kanamycin sulfate (1000 mg/kg, im, St. Louis, MO, USA) [6]. Auditory brainstem response measurements for confirmation of stable long-term hearing loss were performed 3 days after drugs administration, as described previously [7]. Briefly, the scalp electrodes were inserted at the vertex and pinna in anesthetized mice (xylazine, 0.1 mg/kg and ketamine, 30 mg/kg). A series of high-rate 5-ms tone pips and clicks were presented. Levels were incremented in 5 dB steps from 10 to 100 dB SPL. Each click evoked waves of neural activity in the brainstem that were computer-averaged so we could differentiate them from non-auditory background voltages. Both ears were measured.

**The study of lymphatic clearance of FITCD from the brain after OBBB**. 1 h after music-OBBB, FITCD was injected intravenously (1 mg/25 g mouse, 0.5% solution in 0.9% physiological saline, Sigma-Aldrich, St. Louis, USA) and allowed to circulate for 30 min. Afterward, mice were decapitated; their dcLNs were removed and fixed in 4% buffered PFA for one day. To label the lymphatic and blood vessels, samples were incubated overnight at +4C with goat anti-rabbit Lyve-1 and Prox-1 antibody (1:500; Invitrogen, Molecular Probes, Eugene, Oregon, USA). After several rinses in PBS, the samples were incubated for 3h at room temperature with fluorescent-labeled secondary antibodies on 1% BSA/0.2% Triton X-100 /PBS (1:500; goat anti-rabbit IgG (H+L) Alexa Four 555 and goat anti-mouse IgG (H+L) Alexa Four 647; Invitrogen, Molecular Probes, Eugene, Oregon, USA) with further confocal analysis (Olympus FV10i-W, Olympus, Japan).

**Embedding of auditory bulla and capsule in paraffin blocks** was performed using protocol published in Ref. 8. Mice were euthanized with an intraperitoneal injection of a lethal dose of ketamine and xylazine. Then the head was decapitated and the skin was peeled completely towards the nose and cut off together with the snout and incisors. Scissors were inserted into the mouth and the masseter muscles were cut on both sides. The jaw was opened carefully and removed together with the tongue. Using sharp scissors, the skull was skilled into two halves along the midsagittal plane. Cerebral and cerebellar hemispheres with the brainstem were removed. Under a binocular microscope, the bulla and capsule with the surrounding skull bone were dissected. The anterior end of the bulla was cut with scissors and allowed 4% paraformaldehyde (PFA) in PBS to enter into the bulla. Then the bulla and capsule left in the fixative at 4 °C O/N on a tube rotator. Decalcification of the bulla and the capsule was done for

a week at 4 °C in 10% ethylenediaminetetraacetic acid disodium salt dihydrate (EDTA-2Na), 100 mM Tris base, pH 7.0, in a 2 mL tube. Buffer was changed every other day. Afterward, samples were removed and fixed in 4% buffered PFA. Paraformaldehyde-fixed specimens were embedded in paraffin, sectioned (4 µm) and stained with hematoxylin and eosin. Histological sections were evaluated by light microscopy using the digital image analysis system Mikrovizor medical µVizo-103(LOMO, Russia).

**Statistical analysis**. The results are presented as mean ± standard error of the mean (SEM). Differences from the initial level in the same group were evaluated by the Wilcoxon test. Intergroup differences were evaluated using the Mann-Whitney test and ANOVA-2 (post hoc analysis with Duncan's rank test). The significance levels were set at $p < 0.05$-0.001 for all analyses.

## References:


1. Villette V., Chavarha M., Dimov I. et al. Ultrafast Two-Photon Imaging of a High-Gain Voltage Indicator in Awake Behaving Mice. Cell, 179(7), 1590-1608.e23. (doi.org/10.1016/j.cell.2019.11.004)
2. Yisong Qi, Tingting Yu, Jianyi Xu et. al. 2019 FDISCO: Advanced solvent-based clearing method for imaging whole organs. Sci. Adv, 5: eaau8355. (doi: 10.1126/sciadv.aau8355)
3. Bragin D, Kameneva M, Bragina O, Thomson S, Statom G, Lara D, Yang Y, Nemoto E. 2017 Rheological effects of drug-reducing polymers improve cerebral blood flow and oxygenation after traumatic brain injury in rats. *J. Cereb. Blood Flow Metab*. **37**(3), 762-775. (doi: 10.1177/0271678X16684153)
4. Patlak S.C, Blasberg R.G, Fenstermacher J.D. 1983 Graphical evaluation of blood-to-brain transfer constants from multiple-time uptake data. *J. Cereb. Blood Flow Metab*. **3**:1–7. (doi: 10.1038/jcbfm.1983.1)
5. Abdurashitov A, Lychagov V, Sindeeva O, Semyachkina-Glushkovskaya O, Tuchin V. 2015 Histogram analysis of laser speckle contrast image for cerebral blood flow monitoring. *Front. Optoelectron*. **8**(2), 187-194. (doi.org/10.1007/s12200-015-0493-z)
6. Long M, Hai-jin Y, Fen-qian Y, Wei-wei G, Shi-ming Y. 2015 An efficient strategy for establishing a model of sensorineural deafness in rats. *Neural Regen Res*, **10**(10): 1683–1689. (doi: 10.4103/1673-5374.153704)
7. Liberman M, Gao J, He D, Wu X, Jia S, Zuo J. 2002 Prestin is required for electromotility of the outer hair cell and for the cochlear amplifier. Nature, 419:300–304. (doi: 10.1038/nature01059)
8. Sakamoto, A., Kuroda, Y., Kanzaki, S., Matsuo, K. 2017 Dissection of the Auditory Bulla in Postnatal Mice: Isolation of the Middle Ear Bones and Histological Analysis. *J. Vis. Exp*. **11**9, e55054. (doi:10.3791/55054)
